# Associations between upper extremity functioning and kinematics in people with spinal cord injury

**DOI:** 10.1186/s12984-021-00938-9

**Published:** 2021-09-26

**Authors:** Lamprini Lili, Katharina S Sunnerhagen, Tiina Rekand, Margit Alt Murphy

**Affiliations:** 1 Institute of Neuroscience and Physiology, Clinical Neuroscience, Rehabilitation Medicine, Sahlgrenska Academy, University of Gothenburg, Per Dubbsgatan 14, 3rd Floor, 41345 Göteborg, Sweden; 2 Department of Neurology, Haukeland University Hospital, Bergen, Norway; 3 Department of Neurological Rehabilitation, Sahlgrenska University Hospital, Göteborg, Sweden; 4 Department of Occupational Therapy and Physiotherapy, Sahlgrenska University Hospital, Göteborg, Sweden

**Keywords:** Spinal cord injury, Upper extremity, Functioning, Kinematics, Movement analysis, Assessment

## Abstract

**Introduction:**

More knowledge of the relationships between kinematic measures and clinical assessments is required to guide clinical decision making and future research.

**Objectives:**

To determine which kinematic variables obtained during a drinking task were associated with clinical assessments of upper extremity functioning in people with spinal cord injury (SCI).

**Methods:**

In total, 25 individuals with chronic cervical (n = 17) or thoracic (n = 8) complete (n = 14) or motor incomplete (n = 11) SCI (mean age 58.4, SD 13.8) were included. Kinematic data, including movement time, smoothness and joint angles was captured with a 5-camera optoelectronic system during a unimanual drinking task. Action Research Arm Test (ARAT), Sollerman Hand Function Test (SHFT) and basic hand classification of the Upper Extremity Data Set (ISCI-Hand) were used as clinical assessments. Multiple regression analysis was used to identify kinematic variables associated with clinical assessments after controlling for potential confounding factors, such as, age, severity of SCI, sensory function, and hand surgery.

**Results:**

Movement time, smoothness and movement pattern kinematics including trunk displacement, elbow and wrist joint angles were correlated (p < 0.05) with all three clinical scales while the velocity-related kinematics and inter-joint coordination showed low correlations. Multiple regression analysis revealed that wrist angle combined with movement time or smoothness explained 82% and 77% of the total variance in ARAT and SHFT, respectively. Wrist angle alone explained 59% of the variance in ISCI-Hand. The proprioception of the hand increased the explanatory power in the models of ARAT and SHFT. Associations between kinematics and clinical assessments in the subgroup with cervical SCI were equivalent to the whole group analyses. The number of participants in the subgroup with thoracic SCI was small and only allowed limited analysis.

**Conclusions:**

Wrist angle, movement time, movement smoothness are the most important kinematic variables associated with upper extremity clinical assessments in people with SCI. The results are most valid for individuals with cervical SCI. All three assessments are appropriate for SCI. Further research with larger representative sample of thoracic SCI needed.

**Supplementary Information:**

The online version contains supplementary material available at 10.1186/s12984-021-00938-9.

## Introduction

Spinal cord injury (SCI) is a life-changing condition resulting in a partial or complete loss of sensory and/or motor function below the level of injury. About 50% of people with SCI have cervical and 30% thoracic injury [1]. The cervical SCI impacts directly the functioning of upper extremities, although injuries at thoracic level will to some degree also impact upper limb functioning and trunk stability during upper extremity tasks. Upper extremity function plays an important role for person’s autonomy in activities of daily living and quality of life [2, 3]. Upper extremity function and recovery is also one of the highest priorities reported by individuals with SCI [4]. Even though the ability to grasp and manipulate objects predominantly depends on the neurological impairment, compensative strategies, such as, passive tenodesis grasp acquired by learning during rehabilitation or active tenodesis grasp enabled by surgical hand reconstruction, are important for person’s functioning in everyday life [5, 6].

In order to plan and evaluate rehabilitation and other interventions, precise and sensitive assessment of upper extremity functioning after SCI is crucial [7]. The Action Research Arm Test (ARAT) and Sollerman Hand Function Test (SHFT) are two observational performance-based upper extremity activity capacity assessments used in SCI [8–10]. The ARAT measures the same construct as SHFT, but is shorter and more standardized. Standardized collection and reporting of data using Upper Extremity Basic Data Set in SCI populations have also been advocated by the International Spinal Cord Society (ISCoS) [11].

Compared to the traditional clinical assessments, kinematic analysis provides a more objective, precise and sensitive way to measure movement quality and performance during task execution [12]. Kinematic analysis of upper extremity movements during reach-to-grasp tasks is only sparsely investigated in SCI but is particularly important for understanding the movement deficits observed in daily activities [5, 13]. The standardized drinking task is one of the kinematic tasks that is well established [14] and has also been used in individuals with SCI [15].

The relationships between kinematic measures and clinical assessments are important to establish in order to fully use the knowledge of these assessments in clinical decision making. Muscle strength assessment has shown moderate to high correlations with reaching kinematics while correlations with functional independence assessments were weaker in cervical SCI [13]. The impact of sensory function, completeness and severity of the SCI on upper extremity function have been described, although the results vary between studies [16, 17]. Thus, the aim of this study was to determine which kinematic variables obtained during a drinking task were associated with clinical assessments of upper extremity functioning in people with spinal cord injury after controlling for impact of other relevant clinical and demographic factors.

## Material and methods

### Participants

**Fig. 1:**
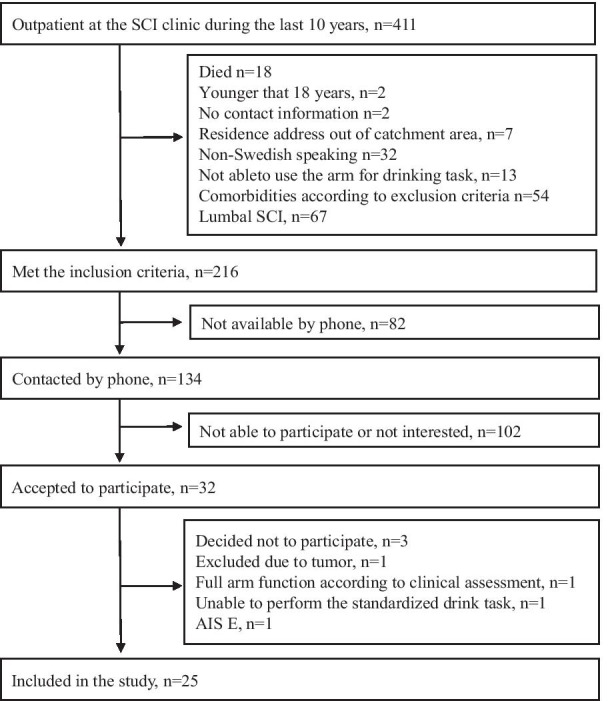
Flow chart over the inclusion process

In this observational cross-sectional study, 25 individuals with complete or incomplete, cervical or thoracic SCI were included. Participants were recruited during 2018 from an outpatient clinic at Sahlgrenska University Hospital (Fig. 1). The inclusion criteria were: age older than 18 years, residence address within the geographical catchment area, having cervical or thoracic SCI injury at least 1 year earlier, impaired sensory and motor function, according to the American Spinal Injury Association (ASIA) Impairment Scale (AIS A, B, C, D) [18], limited upper extremity functioning at least in one arm (< 57 points on ARAT or < 80 points on the SHFT) and ability to perform the standardized drinking task with at least one arm. The exclusion criteria were: unable to communicate in Swedish, other psychological, neurological, musculoskeletal comorbidities that could influence the use of the upper extremity in everyday activities.

The study was approved by the Regional Ethical Review Board in Gothenburg, Sweden (408-17). All participants gave their informed oral and written consent. The study was registered at researchweb.org (https://www.researchweb.org/is/vgr/project/260901) prior to participant enrolment. The reporting of this study conforms to the Strengthening the Reporting of Observational studies in Epidemiology (STROBE) statement [19].

### Clinical assessments

The upper extremity activity capacity, was assessed with ARAT [20] and SHFT [8]. Both arms were assessed, but data only from the more-affected arm was used (except one participant who was able to perform the kinematic drinking task with only one arm and in this case the data from this arm was used). The ARAT assesses unimanual performance, while SHFT includes both unimanual and bimanual tasks. Both scales use ordinal scoring, and consider the time and quality of the observed movement performance.

The ARAT includes 19 items, hierarchically ordered into four subscales (grasp, grip, pinch and gross movement) scored on a 4-points ordinal scale [20, 21]. The sum score varies between 0 and 57, and the higher score indicates better performance. The total administration time is about 5–15 min [20]. The ARAT has shown excellent reliability and validity in stroke [20] and has been increasingly used in people with SCI [9, 10].

The SHFT includes 17 unimanual and 3 bimanual tasks requiring different grips scored on a 5-point scale [8]. The sum score varies between 0 and 80 points and the higher score indicates better performance [8]. The SHFT takes about 20 min to administer [22], but even longer times like 60–90 min have been reported [23]. The SHFT is reliable, valid and recommended for SCI [8, 24].

The hand function was classified according to the International SCI Upper Extremity Basic Data Set by using the “Basic hand—upper extremity function” variable (ISCI-Hand) [11]. The ISCI-Hand uses a 5-level scoring based on the voluntary motor innervation of the upper extremity muscles required to perform common arm and hand movements like grasping, manipulation and arm positioning [11].

### Other clinical characteristics

The neurological level of the injury was classified according to the International Standards for Neurological Classification of Spinal Cord Injury (ISNCSCI/ASIA examination) [18]. The completeness of the injury was assessed according to the ASIA Impairment scale (AIS) [18]. The severity of SCI was classified in five neurologic categories (Table 1) according to International Spinal Cord Injury Core Data Set [25]. The sensory function was assessed by light touch and pin prick in the key sensory points of the hand according the ASIA. Passive motion direction discrimination was used to assess proprioception of the hand. Having undergone hand surgery was also recorded. The independence in self-care was scored by the Spinal Cord Independence Measure (SCIM III) [26].

**Table 1:** Background characteristics of the participants

Characteristics, n = 25	Mean (SD); median (Q1–Q3) or n (%)
Age	58.4 (13.8); 55 (49.5–71)
Sex	
Male	18 (72%)
Female	7 (28%)
BMI	24.8 (4.5); 23.6 (21.6–27.2)
Years since SCI	17.5 (15.4); 9 (5.5–33)
Aetiology of lesion	
Traumatic	20 (80%)
Non-traumatic	5 (20%)
Level of SCI	
Cervical	17 (68%)
Thoracic	8 (32%)
Motor completeness of SCI	
AIS A	10 (40%)
AIS B	4 (16%)
AIS C	3 (12%)
AIS D	8 (32%)
Severity of SCI	
C1–C4 A, B, C	5 (20%)
C5–C8 A, B, C	5 (20%)
T1-S A, B, C	7 (28%)
AIS D	8 (32%)
Hand surgery	8 (32%)
Impaired sensation (tested hand)	18 (72%)
Impaired proprioception (tested hand)	8 (33%)
More-affected arm as dominant	6 (24%)
SCIM III self-care (0–20)	15.1 (5.3); 18 (10.5–19.5)
Action Research Arm Test (0–57)	46.3 (12.9); 52 (37.5–57)
Sollerman Hand Function Test (0–80)	63.0 (20.1); 74 (56–77)
ISCI-Hand (1–5)	
No function (1)	0
Passive tenodesis (2)	2 (8%)
Active tenodesis (3)	3 (12%)
Active extrinsic (4)	4 (16%)
Active extrinsic—intrinsic (5)	16 (64%)

BMI, Body Mass Index; SCI, Spinal Cord Injury; AIS, American Spinal Injury Association (ASIA) Impairment Scale (AIS A, B, C, D); C, Cervical; T, Thoracic; S, Sacral; SCIM, Spinal Cord Independence Measure; ISCI-Hand, basic Hand—upper extremity function according to the International Spinal Cord Injury Upper Extremity Basic Data Set

### Kinematic analysis of the drinking task

A 5-camera optoelectronic motion capture system (Pro Reflex Motion Capture System, MCU240 Hz, Qualisys AB, Gothenburg, Sweden) was used for acquisition of kinematic data. Eight passive spherical markers (12 mm) were attached on the tested hand (third metacarpophalangeal joint), wrist (the styloid process of ulna), elbow (lateral epicondyle), both shoulders (middle part of acromion), thorax (upper part of sternum), face (notch between eyebrows) and drinking glass [27]. Kinematic 3D data from markers was automatically identified and transferred for offline custom-made analysis in the MATLAB^®^ software (The Math Works Inc). Kinematic data was filtered using a 6-Hz second-order Butterworth filter in both forward and reverse directions, resulting in a zero-phase distortion and fourth-order filtering [12, 27]. Detailed procedures for data acquisition (including a video tutorial of the set-up) and analyses of kinematic data can be found in previous publications [12, 27].

The standardized drinking task comprised five movement phases: (i) reaching (reaching and grasping the glass), (ii) forward transport (securing the grasp and transporting the glass to the mouth), (iii) drinking (taking one sip of water), (iv) backward transport (moving the glass back on the table and releasing the grasp), (v) and returning (moving the hand back to the initial position) [12, 27]. Participants were sitting in front of a height adjustable table with approximately 90° knee and hip flexion. The tested hand was resting on the table palm downward with the wrist aligned to the table edge in front of the shoulder. Elbow was positioned in 90° flexion with forearm in horizontal and the upper arm in vertical position. Participants using a wheelchair were sitting in their own chairs. All participants were instructed to sit with their back against the chair back during the entire task, although the trunk movements were not restricted. The hard-plastic drinking glass was filled with 100 ml water and placed 30 cm from the table edge in the midline of the body (about 75% of the arm’s length).

The unimanual drinking task was performed 8–10 times. A mean of all trials performed with the more-affected arm was used in the analysis (one participant was able to perform the drinking task with only one arm). If the participant was unable to use the standard glass, other types of drinking cups were available (hard-plastic wine glass or plastic coffee cup with a handle).

### Kinematic variables

Movement time was calculated for the entire task (total movement time) and separately for each movement phase. The start and end of movement phases were defined by the velocity of the hand marker (2% of the maximum velocity) [12, 27]. The number of movement units (NMU) was computed from the tangential velocity profile of the hand marker for the entire task (excluding the drinking phase) and separately for the first two phases (reaching and forward transport) and for the last two phases (backward transport and returning). A movement unit was defined as a difference between a local minimum and the next maximum velocity value that exceeded the amplitude limit of 20 mm/s, where the time between two subsequent peaks had to be at least 150 ms [12, 27]. NMU captures the repeated sub-accelerations and sub-decelerations during movement performance and can be defined as movement smoothness. The minimum number for movement units for the drinking task is 4 (one unit for each movement phase). The peak hand velocity and the percentage of time to peak velocity in the reaching phase was calculated from the hand marker data. The peak elbow angular velocity during elbow extension in reaching phase was also computed.

The joint angles of the wrist and elbow were determined by the angles between the vectors joining the hand, wrist, elbow and shoulder markers. The shoulder abduction angle was defined as the angle between the vectors joining the shoulder and elbow markers and the vertical vector from the shoulder marker toward the hip. Joint angles were calculated for maximal elbow extension in reaching, maximal wrist angle (dorsal flexion) in reaching and forward transport and maximal angle in elbow flexion and arm abduction during drinking phase. The inter-joint coordination was calculated as a cross-correlation of the shoulder flexion and elbow extension joint angles during the reaching phase. The trunk displacement was defined as the maximal forward displacement of the thorax marker in the sagittal plane from the initial position during the entire drinking task [12].

### Statistical analysis

The statistical analyses were performed using the IBM Statistical Package for Social Sciences™ (SPSS, version 24). Descriptive statistics were calculated for demographic and clinical characteristics of the participants. The level of significance (alpha value) was set to p < 0.05.

Spearman correlation analysis was used to analyse the strength of correlation between kinematic variables and clinical assessments. The kinematic variables showing statistically significant correlation with clinical assessment scales (dependent variables) were considered as potential independent variables to be included in the multiple regression analysis. Spearman correlation coefficients were interpreted as low (less than 0.50), moderate (0.50–0.75), good (0.75–0.90), and excellent (greater than 0.90).

Multicollinearity, defined as r > 0.7, was checked between all potential independent variables. In case of multicollinearity, separate multiple regression models were performed with each variable. Kinematic variables of total movement time and NMU were selected first if significant correlation with clinical scales was observed. If a higher correlation was noted for a specific movement phase or phases, a separate analysis with these variables were conducted. In multiple regression modelling the backward stepwise regression was used. Only the statistically significant kinematic variables were included in the final models. The model assumptions were verified by means of residual analysis, variance inflation factor and predicted probability plots.

The impact of confounding variables was investigated by adding them, one at a time, to every final model. To verify significant confounding effect, the model’s adjusted R-squared (R^2^) and R^2^ change was checked. The confounding variables that were considered were age, sex, level of SCI (cervical, thoracic), severity of SCI, having undergone hand surgery, sensory function and proprioception of the hand (Table 1).

Complementary sub-group analyses following the same procedure of regression analysis as described above were done separately for participants with cervical and thoracic SCI.

## Results

The mean age of the participants was 58.4 years and the majority were men (72%) with traumatic SCI (80%). The severity of the SCI varied across the study group and covered all neurological levels (Table 1). Four participants were not able to grasp the standard drinking glass with one hand and used instead a hard-plastic wine glass (n = 2) or plastic coffee cup with a handle (n = 2). All participants had some level of upper extremity activity limitation according to the inclusion criteria. None of the participants had full score on the SHFT, but in the subgroup of cervical SCI, 3 out of 17 and in the thoracic subgroup, 4 out of 8 had full score on ARAT. The descriptive statistics of the kinematic variables are shown in Additional file 1: Table S1.

The kinematic end-point measures of movement time and smoothness as well as movement pattern measures of trunk, elbow and wrist joint demonstrated statistically significant correlations with all three clinical assessments (Table 2). No significant correlations were observed for the velocity related measures (peak velocity and time to peak velocity), inter-joint coordination, movement time in drinking and returning phase and elbow extension in reaching when considering all included clinical scales (Table 2). Multicollinearity was found between all movement time and NMU measures, and therefore these variables were added to the multiple regression models separately.

**Table 2:** Spearman correlation coefficients calculated between kinematic variables and clinical assessments

Kinematic variables (n = 25)	ARAT	SHFT	ISCI-Hand
Movement time			
Reaching	− 0.41*	− 0.34	− 0.44*
Forward transport	− 0.71**	− 0.77**	− 0.71**
Drinking	− 0.15	− 0.19	− 0.11
Backward transport	− 0.58**	− 0.63**	− 0.55**
Returning	− 0.31	− 0.42*	− 0.36
Movement time, total	− 0.70**	− 0.70**	− 0.67**
Smoothness (number of movement units)			
Reaching and forward transport	− 0.89**	− 0.80**	− 0.76**
Backward transport and returning	− 0.71**	− 0.80**	− 0.61**
Number of movement units, total	− 0.82**	− 0.84**	− 0.75**
Movement velocity and strategy			
Peak hand velocity (reaching)	0.02	0.05	− 0.01
Time to peak hand velocity (reaching)	− 0.04	0.02	0.09
Peak elbow angle velocity (reaching)	− 0.10	0.07	− 0.04
Movement pattern			
Elbow extension (reaching)	0.16	0.07	0.15
Elbow flexion (drinking)	0.66**	0.62**	0.43*
Arm abduction (drinking)	− 0.34	− 0.36	− 0.48*
Wrist angle (reaching and forward transport)	− 0.60**	0.54**	0.55**
Interjoint coordination (reaching)	− 0.09	− 0.20	− 0.14
Trunk displacement	− 0.52**	− 0.50*	− 0.39

ARAT, Action Research Arm Test; SHFT, Sollerman Hand Function Test; ISCI-Hand, basic Hand—upper extremity function according to the International Spinal Cord Injury Upper Extremity Basic Data Set

^**^p < 0.01 *p < 0.05

**Fig. 2:**
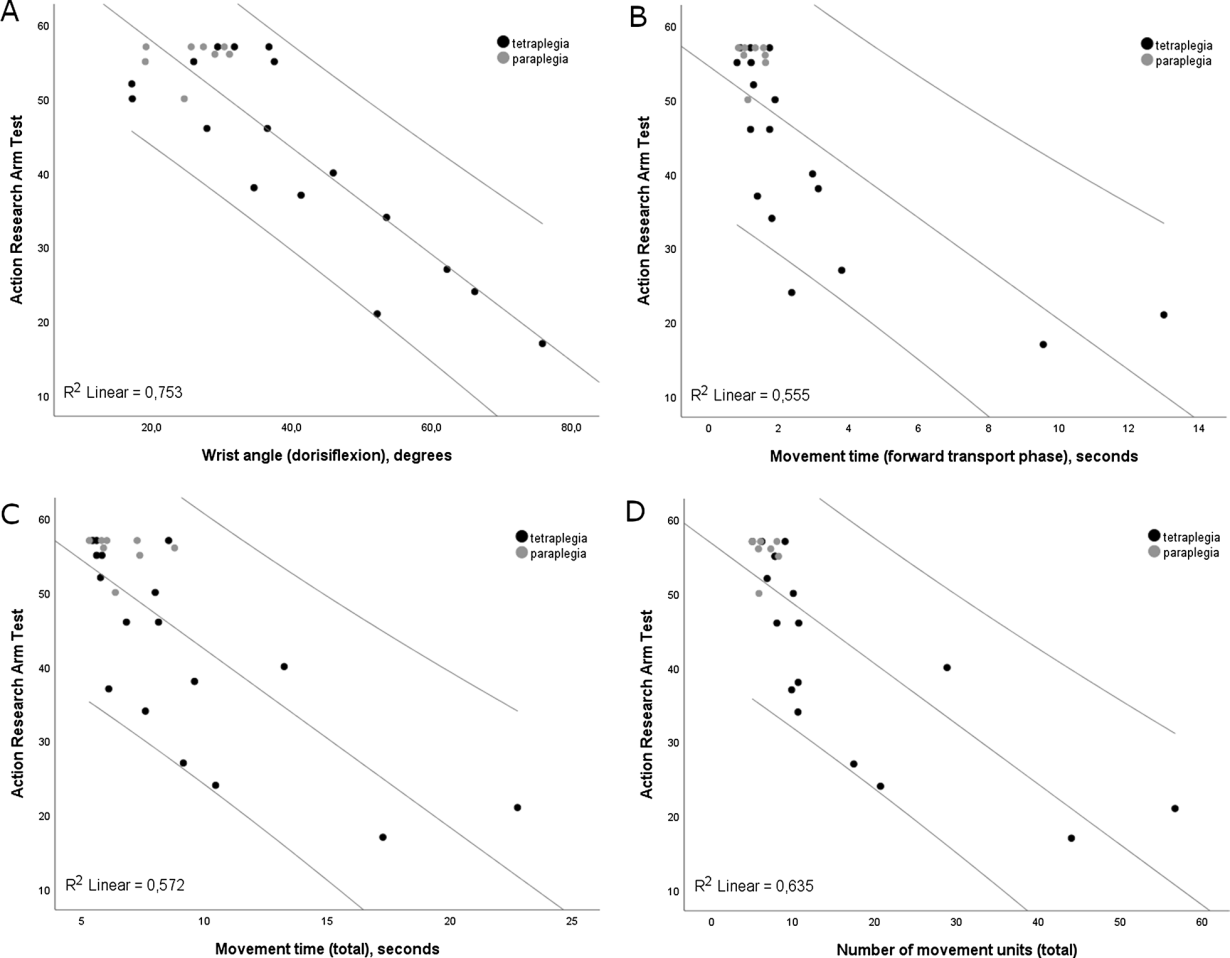
Scatterplots showing correlations and R^2^ values between the Action Research Arm Test and the significant kinematic measures in the final models of multiple regression analyses

All final multiple regression models with ARAT included two variables, wrist angle in combination with forward transport time, total movement time, NMU in the first 2 phases or NMU total, explaining about 82–83% of the total variance in ARAT (Table 3, Fig. 2). In all four models, the wrist angle uniquely explained the largest amount of variance (19–28%). After controlling for confounding variables, only the proprioception of the hand improved (p < 0.01) the explanatory power of the final models up to 90–91%.

**Table 3:** The final models of multiple regression analysis for the whole SCI group (n = 25)

	Estimates of the independent variables				Model statistics	
	Unstand B	Stand B	p-value	Partial correlation (%)	Adjusted R^2^	p-value
Dependent variable ARAT						
Model 1						
MT forward	− 1.61	− 0.35	0.004	8.0	0.83	< 0.001
Wrist angle	− 0.55	− 0.66	< 0.001	27.7		
Model 2						
MT total	− 1.11	− 0.35	0.005	7.5	0.82	< 0.001
Wrist angle	− 0.54	− 0.65	< 0.001	25.6		
Model 3						
NMU total	− 0.39	− 0.38	0.005	7.6	0.82	< 0.001
Wrist angle	− 0.50	− 0.61	< 0.001	19.4		
Model 4						
NMU ReachForw	− 0.58	− 0.39	0.004	8.0	0.82	< 0.001
Wrist angle	− 0.50	− 0.60	< 0.0001	19.1		
Dependent variable SHFT						
Model 1						
MT forward	− 3.59	− 0.50	< 0.001	16.2	0.79	< 0.001
Wrist angle	− 0.65	− 0.50	< 0.001	16.0		
Model 2						
MT total	− 2.44	− 0.49	0.001	14.9	0.79	< 0.001
Wrist angle	− 0.64	− 0.49	0.001	14.7		
Model 3						
NMU total	− 0.86	− 0.54	0.001	15.3	0.77	< 0.001
Wrist angle	− 0.56	0.43	0.004	9.7		
Dependent variable ISCI-Hand						
Model 1						
Wrist angle	− 0.05	− 0.77	< 0.0001	59.3	0.593	< 0.001

ARAT, Action Research Arm Test; SHFT, Sollerman Hand Function Test; ISCI-Hand, basic Hand—upper extremity function according to the International Spinal Cord Injury Upper Extremity Basic Data Set; MT Forward, Movement Time in Forward transport phase; MT Total, Movement Time for the entire drinking task; NMU ReachForw, Number of Movement Units in Reaching and Forward transport phase

**Fig. 3:**
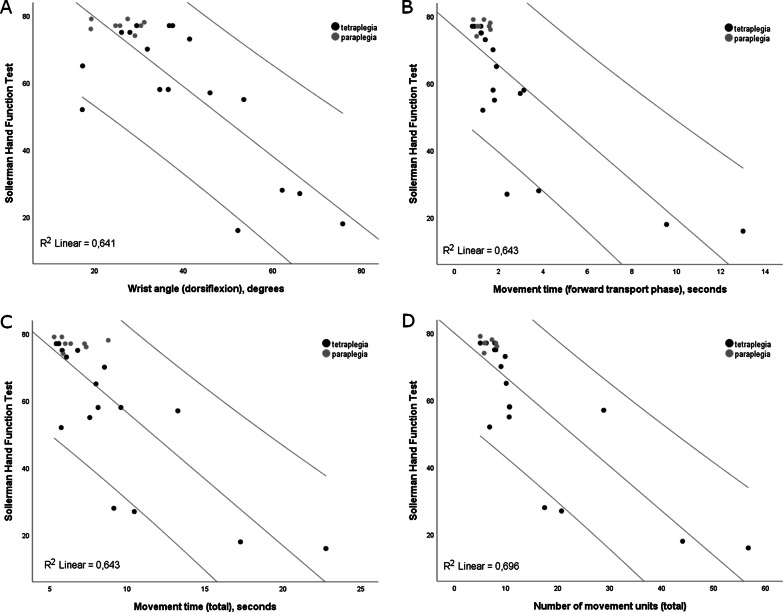
Scatterplots showing correlations and R^2^ values between the Sollerman Hand Function Test and the significant kinematic measures in the final models of multiple regression analyses

The final models with SHFT included wrist angle in combination with forward transport time, total movement time or NMU total, explaining about 77–79% of the total variance (Table 3, Fig. 3). The unique contribution of the included variables in each model was comparable and varied between 10 and 16%. The proprioception of hand was the only confounding variable that improved the explanatory power of the final models (86%).

**Fig. 4:**
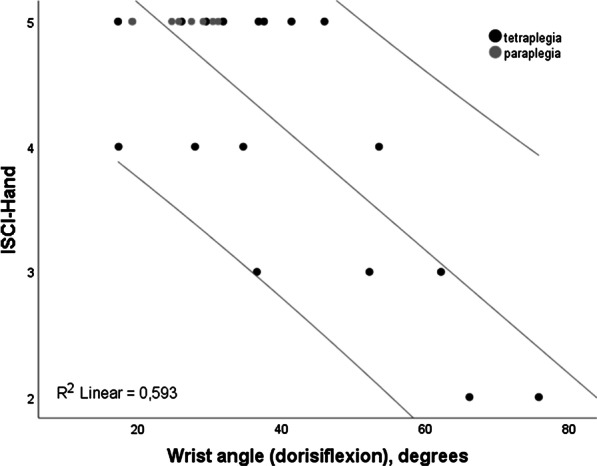
Scatterplots showing correlations and R^2^ values between the ISCI-Hand and the significant kinematic measures in the final models of multiple regression analyses

In the final model with the ISCI-Hand, the wrist angle was the only significant variable, explaining the 59.3% of the total variance (Table 3, Fig. 4). None of the confounding variables influenced the final models significantly.

The complementary sub-group analyses in participants with cervical SCI (n = 17) produced equivalent results to the whole group analysis (Additional file 1: Tables S2 and S3). The subgroup with thoracic SCI was small (n = 8) and lacked statistical power for multiple regression analysis and the results for correlation analysis are considered as uncertain due to the small sample size. In general, the Spearman correlation coefficients were less consistent and lower compared to the cervical SCI group (Additional file 1: Table S4). Moderate correlations (r > 0.50) were observed for movement times, NMU, elbow flexion and inter-joint coordination with the tested clinical scales. All participants with thoracic SCI had an ISCI-Hand score of 5 and therefore correlation analysis was not possible. The range of scores in ARAT (50–57) and SHFT (74–79) covered only the upper end of the scale in the subgroup of thoracic SCI.

## Discussion

This cross-sectional study aimed to determine which kinematic variables obtained during a drinking task were associated with three clinical assessments of upper extremity functioning (ARAT, SHFT, ISCI-Hand) in people with cervical or thoracic spinal cord injury. The multiple regression analysis showed that as for the whole group the wrist angle combined with movement time or movement smoothness explained 82% and 77% or more of the total variance in ARAT and SHFT, respectively. The wrist angle showed the strongest associations with the ARAT, followed by the SHFT, in which the wrist angle contributed equally compared to the movement time and smoothness. The wrist angle was the single kinematic variable associated with the ISCI-Hand explaining about 59% of the total variance. The proprioception was the only variable that significantly improved the total amount of the variance explained by the final models of the ARAT and SHFT. Associations between kinematics and clinical assessments in the subgroup with cervical SCI (n = 17) were equivalent to the whole group analyses. The small number of participants in the subgroup with thoracic SCI (n = 8) did not allow to make specific conclusions regarding the associations in this subgroup. In overall, the findings show that all three clinical assessments, included in this study, reflected well the quality of movement measured with kinematics in individuals with SCI and particularly in those with cervical spinal SCI. Even when there are some differences between these three clinical assessments, all three proved to be appropriate for the assessment of upper extremity functioning.

Our results confirm and extend the previous knowledge from kinematic studies in SCI. A previous study using comparable kinematic analysis of a reach-to-grasp task in individuals with motor complete cervical SCI showed that the ASIA upper extremity motor score was moderately correlated with movement time, movement smoothness and wrist angle [13]. Correlations between kinematics and functional independence in activities of daily life, assessed with SCIM and the motor sections of the Functional Independence Scale, varied but were more consistent with movement smoothness variables [13]. Even though the clinical outcome measures were different in our study, the results show similar pattern. The lower functioning level, assessed with clinical scales, was associated with larger wrist joint angle, slower movement time and increased number of movement units (smoothness). Thus, the end-point measures of movement time and smoothness along with wrist angle demonstrate to be the key kinematic metrics to be considered when evaluating the movement quality and performance in people with cervical SCI.

The strong associations found between clinical assessments and the wrist angle implies that the wrist angle is a key kinematic variable, characterizing movement pattern alterations in people with cervical SCI. Previous kinematic studies have also demonstrated that larger wrist angle is commonly employed by individuals with SCI in reach-to-grasp tasks [15, 28]. A larger wrist joint angle indicates that the tenodesis grasp is used in grasping. This can either be a compensative strategy (passive tenodesis), an effect of upper extremity recovery or a result of hand reconstruction (active tenodesis) [15, 28].

During reaching, the wrist is kept flexed [15, 28] while it is extended during grasping [28] [15] favoring the passive finger-to-palm flexion using the gravity and the passive shortening of flexor digitorum superficialis and profundus. Furthermore, in individuals with SCI, the formation of grasp occurs sequentially after reaching which leads in slower and more segmented upper extremity movement [28]. This phenomenon is in line with our results showing that slower movement time, less smooth movement and increased wrist angle together explained the largest amount of variance in clinical assessments.

The proprioception of the hand improved the explanatory power of the final models with ARAT or SHFT. Even though the exact role of the proprioception is not well-clarified yet [29], the assessment of the proprioception is recommended by ISCoS as an optional element complementary to ISNCSCI/ASIA examination [18]. Our results support this recommendation by showing that clinical assessment of proprioception might add important information on upper extremity functioning in SCI. The precise role of the proprioception needs, however, to be investigated further.

Surprisingly, severity of SCI, having cervical or thoracic SCI, sensory function of the hand or having undergone hand surgery did not influence the explanatory power of the final models. This finding is in line with previous research, suggesting that the components of the ISNCSCI/ASIA examination alone are limited for evaluation of movement performance and need to be complemented with functional assessments [17, 24].

The kinematic analysis confirmed that all three clinical scales used in this study can be used as a proxy to quantify movement deficits in people with SCI. The SHFT was originally developed and validated for evaluation of reconstructive hand surgery after SCI and has been used as a templet and reference standard for development of several other upper extremity scales [8, 23, 30]. The shorter administration time, more standardized scoring and stronger psychometric properties reported for the ARAT support, however, the increased use of ARAT in SCI [9, 10, 17]. In the current study, the ARAT showed the strongest association with kinematic measures compared to the other scales.

About 60% of variance in the ISCI-Hand was explained by the wrist joint angle used during the drinking task. This finding reflects well the nature of the ISCI-Hand scoring, which along with evaluation of voluntary muscle innervation consider the person’s ability to grasp and hold objects either with or without the tenodesis effect [11]. Since the time and movement quality is not considered in the scoring of ISCI-Hand it was not surprising that these kinematic variables were not included in the final multiple regression models.

## Strengths and limitations

This study included a representative sample of individuals with both cervical (68%) and thoracic (32%) SCI evenly distributed across four different grades of severity, which strengthens the generalizability of the results. The sample size was relatively large compared to other studies using kinematic analysis [5], although the subgroup with thoracic SCI was small. Therefore, the results from the current study are most applicable for people with cervical SCI. Future studies with a large sample of thoracic SCI are needed to explore the potential deficits in kinematic parameters and their associations with clinical assessments. It is worth to notice that most of the individuals with thoracic SCI will only have minor remaining impairment or limitation connected to the upper extremity functioning and therefore the clinical assessment needs to be sensitive enough to capture the variations in functioning. For example, the ISCI-Hand classification showed to be too crude for this kind of analysis. Also, as seen in the current study, the ARAT and SHFT might have limited sensitivity to capture smaller variations in functioning in this subgroup with thoracic SCI.

In the current study, three clinical assessments were selected for the analysis. All three, showed associations with kinematics and could therefore be recommended for evaluation of upper extremity functioning in people with SCI. Even though the psychometric properties of these scales have been tested, more studies in SCI populations are needed for prove their psychometric validity. Future studies investigating associations between kinematics and other clinical assessments, commonly used in SCI populations, are also warranted.

In the current study an advanced kinematic optoelectronic motion capture system was used to analyze the specific components of movements. This kind of equipment is commonly not available in clinical settings. However, useful information can be gathered and analyzed that can help the researchers and clinicians to better understand the underlying components of upper extremity functioning.

The results of the current study are only applicable for individuals with SCI who have some limitations in arm functioning but are able to use their upper extremity for drinking from a glass or drinking cup.

## Conclusions

Our results revealed that wrist angle together with movement time and movement smoothness explained most of the variance in upper extremity functioning as measured by clinical scales. Proprioception of the hand contributed also significantly as explanatory variable in the final models, and should therefore be considered in clinical examination. All three clinical assessments, ARAT, SHFT and ISCI-Hand, used in the current study, can be considered to be valid for the assessment of upper extremity functioning after SCI. The selection of a specific scale for use in clinical practice or in research should depend on the specific aim of the assessment, time constraints and expertise of the users. However, in the current study the ARAT showed slightly stronger association with kinematic measures compared to the other scales, which suggest a preference to ARAT in this population. Future studies investigating associations between kinematics and other clinical assessments and studies including a larger representative sample of thoracic SCI are also necessary.

## Supplementary Information

**Additional file 1: Table S1. **Mean and standard deviation of the kinematic variables in all participants with SCI. **Table S2.** Spearman correlation coefficients calculated between kinematic variables and clinical assessments for the subgroup of cervical SCI. **Table S3.** The final models of multiple regression analysis for the subgroup of cervical SCI (n = 17). **Table S4.** Spearman correlation coefficients calculated between kinematic variables and clinical assessments for the subgroup of thoracic SCI (n = 8).

## Data Availability

The data that support the findings of this study are available via corresponding author on a reasonable request.

## Abbreviations

SCI: Spinal cord injury
ARAT: Action Research Arm Test
SHFT: Sollerman Hand Function Test
ISCI-Hand: Basic Hand—upper extremity function variable of the International Spinal Cord Injury Upper Extremity Basic Data Set
ISCoS: International Spinal Cord Society
ASIA: American Spinal Injury Association
AIS: ASIA Impairment Scale
STROBE: Strengthening the Reporting of OBservational studies in Epidemiology
ISNCSCI: International Standards for Neurological Classification of Spinal Cord Injury
SCIM: Spinal Cord Independence Measure
SCIM-III: Spinal Cord Independence Measure version III
SPSS: Statistical Package for Social Sciences
NMU: Number of Movement Units
SD: Standard Deviation
