# Supplementary material for: Associations between upper extremity functioning and kinematics in people with spinal cord injury
Source: J Neuroeng Rehabil. 2021 Sep 26;18:147. doi: 10.1186/s12984-021-00938-9 (PMC8474732; doi:10.1186/s12984-021-00938-9)
Supplement: Supplementary file 1 — Additional file 1: Table S1. Mean and standard deviation of the kinematic variables in all participants with SCI. Table S2. Spearman correlation coefficients calculated between kinematic variables and clinical assessments for the subgroup of cervical SCI. Table S3. The final models of multiple regression analysis for the subgroup of cervical SCI (n = 17). Table S4. Spearman correlation coefficients calculated between kinematic variables and clinical assessments for the subgroup of thoracic SCI (n = 8). [file 12984_2021_938_MOESM1_ESM.docx]

**Table S1.** Mean and standard deviation of the kinematic variables in all participants with SCI.

| **Kinematic variables (n=25)** | **Mean** | **SD** |
| --- | --- | --- |
| **Movement time, seconds** | | |
| Reaching | 1.10 | 0.28 |
| Forward transport | 2.40 | 2.81 |
| Drinking | 1.54 | 0.46 |
| Backward transport | 2.01 | 0.75 |
| Returning | 1.29 | 0.64 |
| Movement time, total | 8.33 | 4.07 |
| **Smoothness (number of movement units), n** | | |
| Reaching & forward transport | 6.48 | 8.69 |
| Backward transport & returning | 6.35 | 4.38 |
| Number of movement units, total | 12.8 | 12.7 |
| **Movement velocity and strategy** | | |
| Peak hand velocity (reaching), mm/s | 621 | 152 |
| Time to peak hand velocity (reaching), % | 0.41 | 0.08 |
| Peak elbow angle velocity (reaching), degrees/s | 97.6 | 43.5 |
| **Movement pattern** | | |
| Elbow extension (reaching), degree | 55.9 | 11.7 |
| Elbow flexion (drinking), degree | 129 | 9.15 |
| Arm abduction (drinking), degree | 42.5 | 23.3 |
| Wrist angle (reaching & forward transport), degree | 35.9 | 15.5 |
| Interjoint coordination (reaching), r | 0.90 | 0.16 |
| Trunk displacement, cm | 5.57 | 4.20 |

**Table S2.** Spearman correlation coefficients calculated between kinematic variables and clinical assessments for the subgroup of cervical SCI.

| **Kinematic variables (n=17)** | **ARAT** | **SHFT** | **ISCI-Hand** |
| --- | --- | --- | --- |
| **Movement time** | | | |
| Reaching | -0.48 | -0.36 | -0.48 |
| Forward transport | -0.82** | -0.88** | -0.71** |
| Drinking | -0.15 | -0.48 | -0.27 |
| Backward transport | -0.55* | -0.60* | -0.50* |
| Returning | -0.42 | -0.45 | -0.44 |
| Movement time, total | -0.76** | -0.81** | -0.69** |
| **Smoothness (number of movement units)** | | | |
| Reaching & forward transport | -0.91** | -0.86** | -0.74** |
| Backward transport & returning | -0.62** | -0.68** | -0.51* |
| Number of movement units, total | -0.86** | -0.82** | -0.75** |
| **Movement velocity and strategy** | | | |
| Peak hand velocity (reaching) | -0.07 | -0.02 | -0.08 |
| Time to peak hand velocity (reaching) | 0.02 | 0.03 | 0.04 |
| Peak elbow angle velocity (reaching) | -0.14 | -0.02 | -0.13 |
| **Movement pattern** | | | |
| Elbow extension (reaching) | 0.40 | 0.27 | 0.27 |
| Elbow flexion (drinking) | 0.54* | 0.44 | 0.26 |
| Arm abduction (drinking) | -0.51* | -0.52* | -0.62** |
| Wrist angle (reaching & forward transport) | -0.74** | -0.55* | -0.55* |
| Interjoint coordination (reaching) | -0.20 | -0.20 | -0.28 |
| Trunk displacement | -0.43 | -0.47 | -0.31 |

**p<0.01 *p<0.05

Abbreviations: ARAT, Action Research Arm Test; SHFT, Sollerman Hand Function Test; ISCI-Hand, Basic hand – upper extremity function according to the International Spinal Cord Injury Upper Extremity Data Set

**Table S3.** The final models of multiple regression analysis for the subgroup of cervical SCI (n=17).

|  | **Estimates of the independent variables** | | | | **Model statistics** | |
| --- | --- | --- | --- | --- | --- | --- |
|  | **Unstand B** | **Stand B** | **p-value** | **Partial Correlation** | **Adjusted R^2^** | **p-value** |
| **Dependent variable ARAT** | | | | |  |  |
| **Model 1** | | | | |  |  |
| MT Forward | -1,52 | -0.37 | 0.017 | 9.4 % | 0.80 | <0.001 |
| Wrist angle | -0.52 | -0.64 | <0.001 | 28.2 % |  |  |
| **Model 2** | | | | |  |  |
| MT Total | -1.08 | -0.38 | 0.018 | 9.2 % | 0.79 | <0.001 |
| Wrist angle | -0.52 | -0.63 | <0.001 | 26.1 % |  |  |
| **Model 3** | | | | |  |  |
| NMU Total | -0.36 | -0.38 | 0.026 | 8.4 % | 0.79 | <0.001 |
| Wrist angle | -0.50 | -0.61 | 0.001 | 21.6 % |  |  |
| **Model 4** | | | | |  |  |
| NMU ReachForw | -0.54 | -0,40 | 0.017 | 9.4 % | 0.80 | <0.001 |
| Wrist angle | -0.48 | -0.59 | 0.001 | 20.0 % |  |  |
| **Dependent variable SHFT** | | | | |  |  |
| **Model 1** | | | | |  |  |
| MT Forward | -3.52 | -0.54 | 0.004 | 20.1 % | 0.74 | <0.001 |
| Wrist angle | -0.58 | -0.45 | 0.012 | 13.8 % |  |  |
| **Model 2** |  |  |  |  |  |  |
| MT Total | -2.46 | -0.54 | 0.005 | 19.1 % | 0.73 | <0.001 |
| Wrist angle | -0.56 | -0.44 | 0.017 | 12.5 % |  |  |
| **Model 3** | | | | |  |  |
| NMU Total | -0.82 | -0.56 | 0.007 | 18.0 % | 0.72 | <0.001 |
| Wrist angle | -0.51 | 0.40 | 0.039 | 9.2 % |  |  |
| **Dependent variable ISCI-Hand** | | | | |  |  |
| **Model 1** | | | | |  |  |
| Wrist angle | -0.05 | -0.72 | 0.001 | 49.1 % | 0.491 | 0.001 |

Abbreviations: ARAT, Action Research Arm Test; SHFT, Sollerman Hand Function Test; ISCI-Hand, Basic hand – upper extremity function according to the International Spinal Cord Injury Upper Extremity Data Set; MT Forward, movement time in forward transport phase; MT Total, movement time for the entire drinking task; NMU ReachForw, Number of Movement Units in reaching and forward transport phase.

**Table S4.** Spearman correlation coefficients calculated between kinematic variables and clinical assessments for the subgroup of thoracic SCI (n=8).

| **Kinematic variables (n=8)** | **ARAT** | **SHFT** |
| --- | --- | --- |
| **Movement time** | | |
| Reaching | 0.26 | 0.05 |
| Forward transport | -0.33 | -0.12 |
| Drinking | **-0.68** | 0.01 |
| Backward transport | -0.29 | -0.03 |
| Returning | 0.04 | **-0.53** |
| Movement time, total | **-0.51** | -0.36 |
| **Smoothness (number of movement units)** | | |
| Reaching & forward transport | **-0.52** | -0.49 |
| Backward transport & returning | -0.21 | **-0.56** |
| Number of movement units, total | -0.33 | **-0.51** |
| **Movement velocity and strategy** | | |
| Peak hand velocity (reaching) | 0.14 | -0.17 |
| Time to peak hand velocity (reaching) | -0.47 | -0.09 |
| Peak elbow angle velocity (reaching) | -0.28 | 0.12 |
| **Movement pattern** | | |
| Elbow extension (reaching) | -0.12 | -0.06 |
| Elbow flexion (drinking) | **0.60** | **0.57** |
| Arm abduction (drinking) | -0.17 | -0.41 |
| Wrist angle (reaching & forward transport) | 0.23 | 0.06 |
| Interjoint coordination (reaching) | -0.05 | **-0.60** |
| Trunk displacement | -0.49 | 0.16 |

Correlation coefficients should be interpreted as preliminary due to the small sample size.

Abbreviations: ARAT, Action Research Arm Test; SHFT, Sollerman Hand Function Test
